# Supplementary material for: Complexome profiling of the Chlamydomonas psb28 mutant reveals TEF5 as an early PSII assembly factor
Source: Plant Cell. 2025 Mar 17;37(6):koaf055. doi: 10.1093/plcell/koaf055 (PMC12164590; doi:10.1093/plcell/koaf055)
Supplement: koaf055_Supplementary_Data [file koaf055_supplementary_data.zip › psb28_interactive.zip!/plots/intensity/qualityPlots/peptides/ATS2_Cre02.g107450.t1.2.html]

### Cre02.g107450.t1.2

Trivial name: ATS2  
SeqCoverage [median]: 12.60  
Mass: 47.57 kDa  
  
 

| Sequence | MissedCleavage | Mass [Da] | wtIntensity | muIntensity | Score |
| --- | --- | --- | --- | --- | --- |
| AELNNPNIK | 0 | 1011.53 | 920.66 | 1014.27 | 85.21 |
| SVVSVLR | 0 | 758.47 | 0.00 | 691.22 | 81.78 |
| TSSLEHPGVQMVAQER | 0 | 1767.86 | 0.00 | 745.61 | 60.63 |
| YHTYEVLK | 0 | 1051.53 | 0.00 | 275.35 | 52.79 |
| YNGQDLGVLEVESK | 0 | 1549.76 | 0.00 | 516.69 | 63.73 |
|  |  |  | 920.7 | 3243.1 |  |
